# Transfer with microbiota from lean donors prevents excessive weight gain and restores gut-brain vagal signaling in obese rats maintained on a high fat diet

**DOI:** 10.21203/rs.3.rs-4438240/v1

**Published:** 2024-05-31

**Authors:** Dulce M. Minaya, Jiyoung S. Kim, Rebecca Kirkland, Jillian Allen, Sitara Cullinan, Neil Maclang, Guillaume de Lartigue, Claire B de La Serre

**Affiliations:** a Department of Nutritional Science, University of Georgia, Athens, GA; b Emory University School of Medicine, Atlanta, GA; c Monell Chemical Senses Center, Philadelphia, PA; d Department of Biomedical Sciences, Colorado State University, Fort Collins, CO

**Keywords:** Gut microbiota, Microbiota Transfer, Gut-brain axis, Dysbiosis, Obesity

## Abstract

**Background.:**

The collection of microorganisms, mainly bacteria, which live in the gastrointestinal (GI) tract are collectible known as the gut microbiota. GI bacteria play an active role in regulation of the host’s immune system and metabolism, as well as certain pathophysiological processes. Diet is the main factor modulating GI microbiota composition and recent studies have shown that high fat (HF) diets induce detrimental changes, known as dysbiosis, in the GI bacterial makeup. HF diet induced microbiota dysbiosis has been associated with structural and functional changes in gut-brain vagally mediated signaling system, associated with overeating and obesity. Although HF-driven changes in microbiota composition are sufficient to alter vagal signaling, it is unknown if restoring normal microbiota in obesity can improve gut-brain signaling and metabolic outcomes. In this study, we evaluated the effect of lean gut microbiota transfer in obese, vagally compromised, rats on gut-brain communication, food intake, and body weight. Male Sprague-Dawley rats were maintained on regular chow, or 45% HF diet for nine weeks followed by three weeks of microbiota depletion using an antibiotic cocktail. The animals were then divided into four groups (n=10 each): **LF** – control group on regular chow, **LF-LF** – chow fed animals that received antibiotics and microbiota from chow fed animals, **HF-LF** – HF fed animals that received microbiota from chow fed animals, and **HF-HF** – HF fed animals that received microbiota from HF fed animals. Animals were gavaged with donor microbiota for three consecutive days on week one and once a week thereafter for three more weeks. HF-LF animals received inulin as a prebiotic to aid the establishment of the lean microbiome.

**Results.:**

We found that transferring a LF microbiota to HF fed animals (HF-LF) reduced caloric intake during the light phase when compared with HF-HF rats and prevented additional excessive weight gain. We did not observe significant changes in the density of vagal afferents terminating in the brainstem among the groups, however, HF-LF animals displayed an increase in postprandial activation of both primary sensory neurons innervating the GI tract and brainstem secondary neurons.

**Conclusions.:**

We concluded from these data that normalizing microbiota composition in obese rats improves gut-brain communication and restores normal feeding patterns which was associated with a reduction in weight gain.

## 1. Introduction

In the United States, 42.4% of the adult population was obese in 2017–2018 (1). Obesity increases the risk of developing cardiometabolic diseases, such as diabetes and hypertension, as well as psychological disorders, and represents a financial burden with an estimated $260.6 billion spent on health care costs (2). Most importantly, obesity and its associated comorbidities have a significant negative impact on the perceived quality of life (3).

It is generally accepted that obesity results from an disparity in energy intake compared to energy expenditure (4). Energy metabolism in the human body is a highly complex, multifactorial process (4). The collection of microorganisms that inhabit the gastrointestinal tract (GI), known as the gut microbiota, plays a critical role in regulating host energy metabolism, thus contributing to metabolic health (5). Obesity is associated with deleterious changes in gut microbiota composition, or dysbiosis (6). Members of the Firmicutes and Bacteroidetes phyla make up ~80% of the gut microbiome, and studies have shown, in humans and rodents, that in obesity the relative abundance of Firmicutes increases while Bacteroidetes decreases (7–11). Similar changes are observed in response to HF feeding (11). Independent of length of dietary intervention, HF diet consumption leads to microbiota dysbiosis marked by a decrease in microbial diversity and increased pro-inflammatory potential of the gut microbiota associated with impaired GI permeability (12, 13). Colonization of germ-free (GF) animals with microbiota from obese or HF fed donors induces an obese phenotype in the conventionalized animals characterized by increased caloric intake and body fat accumulation (14, 15).

Given the role of vagal signaling in regulation of intake and the distribution of vagal terminals along the gastrointestinal lamina propria, it is likely that changes in microbiota composition affect vagal signaling since vagal afferent neurons (VAN) express receptors for bacterial byproducts (16, 17). The vagus nerve provides bidirectional communication between peripheral organs and the brain. Within the GI tract, mechanosensitive vagal sensory terminals respond to distension, and chemosensitive vagal afferent terminals respond to nutrients to suppress meal size (18). Postprandial signals increase neuronal activity in the nucleus tractus solitarius (NTS), the central site of vagal sensory termination (19). Satiety peptides, cholecystokinin (CCK) and glucagon-like peptide 1 release (GLP-1) activate vagal afferents and go into the circulation to signal meal termination and induce satiety (20). Cocaine- and amphetamine-regulated transcript (CART) neuropeptide expression is induced in the NG upon feeding and this response is mediated by CCK (21, 22). CART and CCK-mediated vagal signaling is further associated with c-Fos expression, a marker of neuronal activation, in the NTS and other central areas involved in the control of food intake (23). HF feeding and/or obesity have been shown to reduce VAN sensitivity to tension, nutrients, and GI hormones as well as postprandial NTS activation. These changes have been linked to food overconsumption (24–35). In addition to homeostatic mechanisms, gut-innervating VAN project to limbic brain regions, and this gut-reward circuit is also critical for meal termination (36).

In addition to alteration in vagal function, our lab has previously shown that chronic HF feeding alters vagal structure, causing withdrawal of vagal c-fibers from the NTS. The timing of NTS vagal withdrawal coincides with onset of weight gain, hyperphagia, and insensitivity to GI CCK (12, 13, 37, 38). Nerve injury-induced or diet-induced vagal withdrawal can be followed by NTS reinnervation (sprouting). The progression time for vagal remodeling is dependent on the type of diet, with more energy dense diets causing more rapid progression, suggesting that HF diet consumption triggers a dynamic structural remodeling in the vagal system. It is unclear whether functionality improves along with density. In rats, reinnervation does not appear to fully restore function, as animals remain hyperphagic and insensitive to CCK (13, 39).

Critically, transfer of microbiota from HF fed rats, a dysbiotic microbiota, into germ-free (GF) or microbiota- depleted rats significantly increased recruitment of immune cells in the nodose ganglia (NG, where VAN cell bodies are located) and NTS as well as decreased the density of vagal afferents innervating the NTS. Microbiota-depleted rats colonized with a HF-type microbiota also display reduced sensitivity to the GI satiety peptide CCK (37) and dampened food -associated reward (15). Conversely, normalizing microbiota composition or inhibition of immune cells in HF rats prevented the loss of vagal innervation at the level of the NTS and preserved CCK sensitivity (13, 40). These data show that HF-driven changes in microbiota composition are necessary and sufficient to alter vagal signaling.

It is, however, unknown whether restoring normal microbiota in obesity and/or following diet-driven alterations in vagal signaling can improve gut-brain signaling and promote weight loss. Weight loss in humans and rodents is associated with changes in microbiota composition (41, 42); but there is little evidence that restoring normal microbiota in obesity can improve metabolism. There is some circumstantial evidence in humans that probiotics use is associated with weight loss (43), but no causal relationship has been established. This is partially because in most studies, microbiota manipulations strategies (antibiotics, probiotics) are generally introduced concomitantly to HF feeding (44–47). While these models provide insights into the etiology of obesity, they do not address whether targeting the microbiota in an obese model can modulate body weight. Thus, the focus of this study was to evaluate the effect of gut microbiota transfer in obese, vagally compromised, rats on gut-brain communication, food intake and body weight, 24-h feeding patterns, and willingness to work for a food reward. We found that transferring lean microbiota to HF-fed, obese animals prevented excessive weight gain, normalized feeding patterns, improved learning, and rescued vagally mediated signaling. To our knowledge, this is the first study that has demonstrated that normalizing the gut microbiota composition, independent of diet, can ameliorate some of the detrimental consequences of obesity.

## 2. Methods

### 2.1 Animals

Male Wistar rats (~200 g; Envigo, Indianapolis, IN) were housed individually in conventional polycarbonate shoe-box cages in a temperature-controlled vivarium with *ad libitum* access to regular chow pellets (5% fat, 3.70% sucrose; Laboratory rodent diet, product #5001, Fort Worth, TX) and water. Rats were maintained on a 12:12-h light: dark cycle with lights on at 0100-h and allowed to acclimate to laboratory conditions for one week prior to starting experiments. All animal procedures were approved by the University of Georgia Institutional Animal Care and Use Committee and conformed to the National Institutes of Health Guidelines for the Care and Use of Laboratory Animals.

Following the acclimation period, animals were divided into low fat (LF) (n = 30) and high fat (HF) (n=26) and remained on their respective diets for the duration of the study. The HF group was fed a diet containing 45% kcal from fat (Research Diets D12451, New Brunswick, NJ), and the LF group was maintained on regular chow pellets. All animals had *ad libitum* access to food and water. After nine weeks, the LF group was further split into: donors (DLF, n=10), LF fed controls (LF, n=10), and LF fed and recolonized with LF diet type microbiota (LF-LF, n=10). Similarly, the HF group was divided into: donors (DHF, n=6), HF fed recolonized with a LF type microbiota (HF-LF, n=10), and HF fed and recolonized with HF type microbiota (HF-HF, n=10). At this point, the donor groups were sacrificed to obtain their cecal and fecal samples.

**Figure 1.**
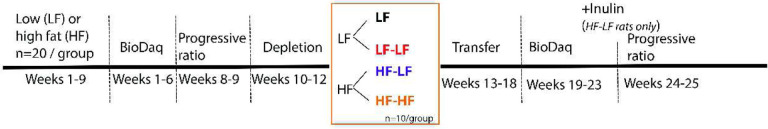
Experimental Timeline Alpha value for statistical significance was set at 0.05.

Starting at week ten of the study, the LF-LF, HF-LF, and HF-HF groups were gavaged daily for 3 days with an antifungal (Amphotericin-B, 1 mg/kg BW; Gold Biotechnology, St. Louis, MO), followed by 15 days of daily gavaging with an antibiotic cocktail consisting of ampicillin, gentamicin, neomycin (100 mg/kg BW, Gold Biotechnology, Olivette, MO), vancomycin (50 mg/kg BW, Gold Biotechnology, Olivette, MO) and metronidazole (100 mg/kg BW, MP-Biomedical LLC., Santa Ana, CA). Flexible tubing (polyethylene O.D. 2.42 mm) connected to a blunt 16 G needle was used for gavaging to minimize discomfort and irritation (48). Following completion of the depletion paradigm, LF-LF, HF-LF, and HF-HF rats were recolonized via oral gavage with fecal/cecal inocula from LF or HF donors. Receiver rats were gavaged with inocula once daily for three consecutive days during the first week followed by once a week for an additional five weeks. The inocula were prepared with 30 μg intestinal matter from the donor rats diluted in 500 μl 20% glycerol in phosphate buffered saline (PBS) (37). Recolonized and control rats had *ad libitum* access to their respective diet and water. Inulin, a known prebiotic, was provided in water at a dose of 100g/10ml to the HF-LF cohort starting at week five of microbiota transfer as it has been shown to aid the establishment of a healthy gut microbiota (49). Body weight and food intake were measured daily. The animals were euthanized 12 weeks after recolonization (Fig. 1).

### 2.2 Behavioral testing

#### 2.2.1 Progressive ratio

Rats were trained to lever press for a 45 mg sucrose or fat pellet reward (product # F0023 and F05989, respectively; Bio-Serv, Flemington, NJ). Standard operant conditioning chambers (Med Associates, St. Albans, VT) equipped with two levers located on either side of a food trough were used for training and testing. A cue light was illuminated above each lever to signal when food pellets were dropped into the trough from a dispenser outside the cage. A computer running custom programs in Med-PC IV software recorded responses and controlled pellet delivery during each session. Each rat was shaped to lever press for reinforcement at a fixed ratio (FR) 1, where the rat received a reward after each lever press. Once stable responding was observed at FR1, rats were tested on a criterion of five consecutive FR3 responses within a 20 min period. After this criterion was achieved, the rats were tested on a criterion of five consecutive FR5 responses within 20min. After reaching this criterion, rats were tested on the following progressive ratio (PR) schedule:1, 2, 4, 6, 9, 12, 15, 20, 25, 32, 40, 50, 62, 77, 95, 118, 145, and 178. The breakpoint was defined as the highest ratio schedule completed by the rats on the PR schedule. Sessions ended if the animal failed to earn a pellet within 20 min or after 2-h.

#### 2.2.2 Feeding pattern analysis

A BioDaq food monitoring system, which allowed uninterrupted recording of individual meals for each animal over several consecutive days, was used to assess meal patterns. Meals were defined by at least 0.1g of food consumed without interruption and the inter-meal interval was set at 15 min.

### 2.3 Microbiota analysis

Fecal samples were collected at the end of the study. Bacterial DNA was extracted from feces using a commercial kit (Quick-DNA Fecal/Soil Microbe Miniprep Kit, cat #D6010, Zymo research, Irvine, CA). High-throughput sequencing was performed using Illumina MiSeq paired-end runs (Georgia Genomics and Bioinformatics Core, Athens, GA). Amplification targeted the V3–V4 region of the 16 S ribosomal RNA genes using the 515F/806R primer set. Bacterial 16S were processed with a DADA2 pipeline. OTUs were assigned to taxa through the Greengenes database. Sequences were subsequently trimmed, joined, and quality filtered. To identify Operational Taxonomic Units (OTUs) and to evaluate beta and alpha diversities, bacterial abundance was normalized by log-transformation. β-diversity ((dis-)similarities between samples) was assessed via Principal Coordinates Analysis (PCoA) with distances determined using the Bray-Curtis index. Significant dissimilarities between groups were determined via permutational multivariate ANOVA (PERMANOVA, MicrobiomeAnalyst) (50).

### 2.4 Euthanasia

After a 12h fast, animals received ~14.28 kcal of their respective diets, were allowed to refeed for 30min, and were sacrificed 90 minutes post meal. Rats were anesthetized with CO_2_ and transcardially perfused with 0.1 M phosphate-buffered saline (PBS; pH 7.4) followed by 4% paraformaldehyde. Hindbrains and nodose ganglia were harvested, postfixed in 4% paraformaldehyde for 2-h, and immersed in 30% sucrose, 0.1% NaN3 in PBS overnight for cryoprotection. Tissues were then stored at −80°C until processing.

### 2.5 Immunofluorescence

Hindbrains and NG were cryosectioned (20 μm and 12 μm, respectively) using a Leica CM1900 cryostat. Sections from the hindbrain were collected from the caudal to the rostral region of the NTS (between bregma −14.16 and −12.93 mm). A subset of animals from each group was used for immunostaining.

Standard immunofluorescence was used to determine the presence of Cocaine and amphetamine-regulated transcript (CART) in the NG, c-Fos immunoreactivity, immune cell activation, and vagal afferent density in the hindbrain. NG sections were incubated overnight with primary antibody against CART (1:200 dilution; Phoenix Pharmaceuticals, cat# H-003–62) followed by Alexa-488 secondary antibody for 2 h. Hindbrain sections were incubated overnight with a primary antibody against either c-Fos (Cell Signaling, cat# 2250) or ionized calcium binding adaptor molecule 1 (Iba1, Wako, Cat# 019–19741, RRDI: AB_839504) followed by Alexa-488 secondary antibody for 2h at room temperature. In addition, hindbrain sections were incubated with GSL I - isolectin B4 biotin-conjugated (IB4, Vector Laboratories Cat# B-1205, RRDI: AB_2314661) overnight followed by ExtrAvidin-CY3 (Sigma-Aldrich Cat# E-4142) for 2 h to visualize unmyelinated c-fibers. Images of the NTS were taken at 10X or 20X, and NG were taken at 20X using a Keyence BZ-X800 (Keyence Corporation of America, Itasca, IL). ImageJ (US National Institutes of Health, Bethesda, MD) was used to quantify the percentage of positive pixels and neuronal cell counts within the region of interest (ROI).

### 2.7 Statistical analysis

GraphPad Prism 7 (GraphPad Software, Inc.) was used to conduct statistical analyses, except when noted otherwise. Data are expressed as mean ± SEM and were analyzed using t-test, ANOVA, or mixed-effects analysis followed by appropriate post-hoc test for multiple comparisons.

## Results

### LF microbiota transfer prevented excessive weight gain in HF fed rats.

**Figure 2.**
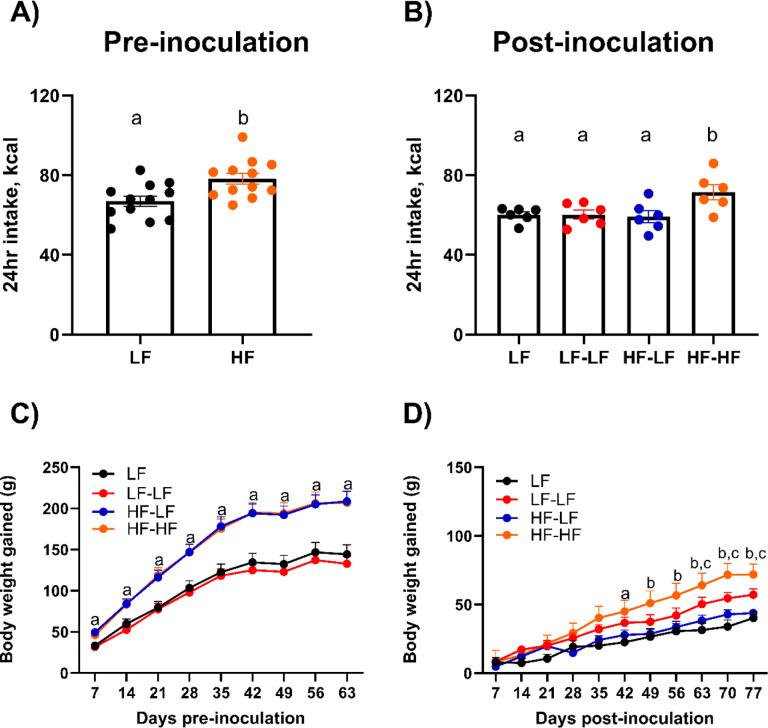
LF microbiota transfer prevented excessive weight gain in HF fed rats. Pre-inoculation caloric intake (**A**, n = 12 per group), pre-inoculation body weight gain (**C**, n = 10 per group), post-inoculation caloric intake (**B**, n = 6 per group), and post-inoculation body weight gain (**D**, n = 10 per group). Pre-inoculation, HF fed rats ate significantly more (p<0.01) and gained significantly more body weight than LF fed animals (p<0.0001). Post-inoculation, HF-HF rats ate significantly more than all other groups (ps<0.05). LF and HF-LF rats gained significantly less body weight than HF-HF rats (ps<0.05). Bars denoted with the same letter are not statistically different. In graphs C and D, ^a^ denotes statistical significance between LF and HF rats, ^b^ denotes statistical significance between LF vs HF-HF and HF-LF vs HF-HF, and ^c^ denotes statistical significance between LF and LF-LF. **LF**: Low fat fed control; **LF-LF**: Low fat rats that received microbiota from low fat fed donors; **HF-LF**; High fat fed rats that received microbiota from low fat fed donors; **HF-HF**: High fat fed rats that received microbiota from high fat fed donors.

Initial body weights were similar for all rats, and they were randomly assigned to either chow or HF diet. 24-hr caloric intake measured using the Biodaq system in a subset of animals from each group and body weights are shown in Fig. 2. Prior to microbiota transfer, HF feeding led a significant increase in daily caloric intake compared to chow (t-test, p<0.01) (Fig. 2A). Consequently, HF fed rats gained significantly more body weight than LF rats (Mixed-effects model, F (3, 36) = 10.67; p<0.0001) (Fig. 2C).

Following microbiota transfer, HF-HF rats had a significantly higher daily intake than all other groups (One-way ANOVA, ps<0.05). There was a significant effect of time (Mixed-effects model, F (3.375, 100.6) = 144.1; p<0.0001) and diet (F (3, 32) = 4.08; p<0.05) on weekly body weight gain as well as a significant interaction (F (30,298) = 4.648; p<0.0001) (Fig. 2D). Following recolonization, HF-LF animals gained significantly less body weight than HF-HF animals (p<0.05) and the rate of body weight gain in the HF-LF group was comparable to that of LF controls. Since HF-LF animals were maintained on a HF diet, inulin was added to their water as a prebiotic to aid the effectiveness of the microbiota transfer (51). Inulin intake did not affect water intake in the HF-LF group compared to the HF-HF group (**Suppl. Fig. 1**).

### LF microbiota transfer improved microbiota profile in HF fed rats.

**Figure 3.**
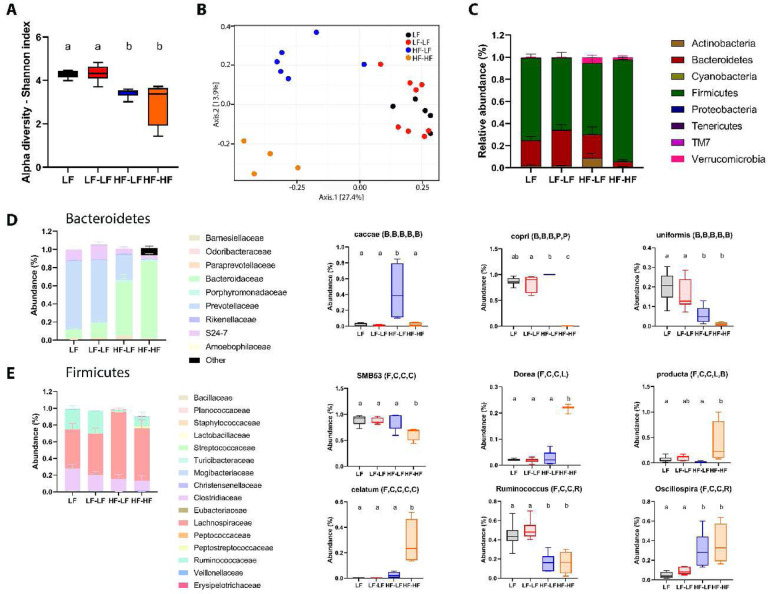
LF microbiota transfer improved microbiota profile in HF fed rats. **A**. Shannon index shown as mean ± SEM for each group. HF fed rats had significantly lower species diversity than LF fed rats (F (3, 19) = 9.741, p<0.001). **B**. Principal coordinate analysis was analyzed using a pairwise PERMANOVA test with Benjamin-Hochberg procedure for multi-testing adjustment. Results revealed significant differences among the groups (F = 5.0392; R^2^=0.4431; p<0.001). The microbiota of LF and LF-LF rats clustered together (P = 0.121) and away from HF-HF (ps<0.01). The microbiota of HF-LF was significantly different from the microbiota of LF and LF-LF (ps<0.01) and HF-HF (p<0.0045) rats. However, it clustered closer to the microbiota of the LF and LF-LF groups than to the HF-HF cohort. **C**. Bacterial phyla abundance was quantified in fecal samples. Bacteroidetes and Firmicutes were the most abundant bacterial phyla in all groups. LF (LF and LF-LF) fed animals had significantly higher abundance of Bacteroidetes than HF (HF-LF and HF-HF) fed rats (Ps < 0.05). However, LF fed animals and HF-LF rats had significantly higher abundance of Bacteroidetes than HF-HF rats (Ps < 0.001). In contrast, HF-HF animals had significantly higher abundance of Firmicutes compared to LF fed and HF-LF rats (Ps < 0.001). **D-E**. Examples of taxa that displayed significantly different patterns of abundance among the groups. Bars denoted with the same letter are not statistically different. **LF (n=5)**: Low fat fed control; **LF-LF (n=8)**: Low fat rats that received microbiota from low fat fed donors; **HF-LF (n=6)**; High fat fed rats that received microbiota from low fat fed donors; **HF-HF (n=4)**: High fat fed rats that received microbiota from high fat fed donors.

Analysis of fecal microbiota composition is shown in Fig. 3. Species richness post inoculation was evaluated using the Shannon index. HF animals had significantly lower species richness compared to LF animals (One-way ANOVA, F (3, 19) = 9.741, p<0.001) (Fig. 3A), regardless of microbiota transplant. We did not observe differences in species evenness. A PERMANOVA analysis of (dis-)similarities between samples revealed a clear separation between the fecal microbiota of LF and HF rats (F = 5.0392; p<0.001) (Fig. 3B). There was no difference between LF and LF-LF groups (p=0.121) as microbiota profiles from these groups clustered together. However, within the HF animals, HF-LF and HF-HF animals displayed significantly different microbiota profiles (p<0.01). HF-LF animals clustered closer to the LF (LF and LF-LF) animals compared to the HF-HF group, yet their microbiota profile did not fully normalize (ps<0.05). Analysis of relative taxa abundance at the phylum level identified significant differences in the Bacteroidetes and Firmicutes phyla among the groups (Two-way ANOVA, F (7, 146) = 412.7; p<0.0001) (Fig. 3C). HF-HF animals exhibited a significant decrease in relative abundance of members of the Bacteroidetes phylum compared to all other groups (Ps<0.001) as well as significant increase in the relative abundance of Firmicutes compared to all other groups (ps<0.0001). HF-LF phyla profile was similar to the LF and LF-LF animals, with no differences in relative abundances between the groups.

Belonging to the Bacteroidetes phylum, the species *caccae* and *copri* were found to be enriched in the HF-LF group compared to the other groups (One-way ANOVA, *caccae*, ps<0.01; copri, ps<0.05 vs. LF-LF and HF-HF). Conversely, the species *uniformis* was not transferred to HF-LF animals as we found a significantly higher abundance of the species in LF compared to HF animals (One-way ANOVA, ps<0.05) (Fig. 3D). Within the Firmicutes phylum (Fig. 3E), species belonging to the SMB53 genus were found to be enriched in LF (LF and LF-LF) compared to the HF-HF animals (One-way ANOVA, ps<0.05) and this trait was successfully transferred to the HF-LF group (HF-LF vs. HF-HF, One-way ANOVA, p<0.05) In addition, Species belonging the genus *Dorea*, and the species *producta* and *celatum* were present in significantly lower abundance in the LF groups (LF and LF-LF) compared to HF-HF animals (One-way ANOVA, ps<0.05) and again, this trait was successfully passed to the HF-LF group (HF-LF vs. HF-HF, p<0.05). Other Firmicutes abundances differed based on diet regardless of microbiota transfer, species of the *Ruminococcus* genus were found to be significantly enriched in LF compared to HF animals (One-way ANOVA, ps<0.001) while species of the genus *Oscillospira* were significantly depleted in LF compared to HF animals (One-way ANOVA, ps<0.05). Overall, the HF-LF group displayed a unique microbiota profile with similarities with both the LF and HF groups, showing the FMT improved but did not fully normalize the animals’ microbiota profile.

### LF microbiota transfer normalized feeding patterns and improved acquisition time for an operant task in HF fed rats.

**Figure 4.**
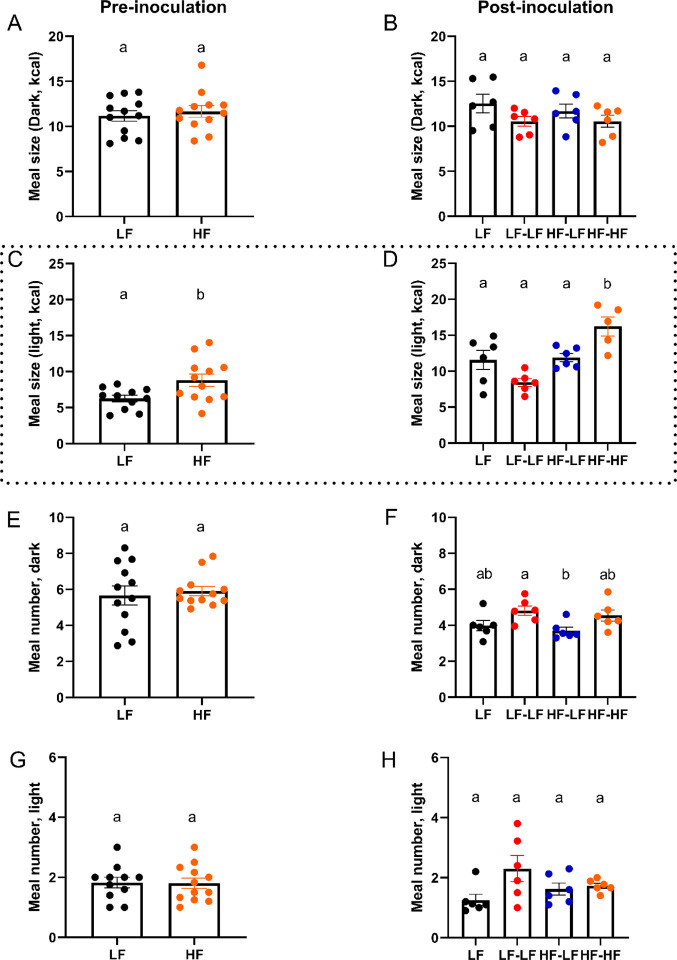
LF microbiota transfer normalized feeding patterns in HF fed rats. This figure shows representative data of 24-h food intake. Pre-inoculation data is shown on the left column. Post-inoculation data is shown on the right column. **A, B**. Meal size during the dark phase. There were no differences in meal size among the groups. **C, D**. Meal size during the light phase. Pre-inoculation, HF feeding significantly increased meal size. Post-inoculation, HF-HF rats had significantly larger meal size compared to LF, LF-LF, and HF-LF. **E, F**. Meal number during the dark phase. Pre-inoculation, there were no differences in meal number between LF and HF fed animals. Post-inoculation, HF-LF animals had a significantly lower meal number than LF-LF rats. **G, H**. Meal number during the light phase. There were no significant differences in meal number during the light phase among the groups. Bars denoted with the same letter are not statistically different. Pre-inoculation, **LF** n=11–12, **HF** n=12. Post-inoculation, **LF (n=6)**: Low fat fed control; **LF-LF (n=6)**: Low fat rats that received microbiota from low fat fed donors; **HF-LF (n=6)**; High fat fed rats that received microbiota from low fat fed donors; **HF-HF (n=6)**: High fat fed rats that received microbiota from high fat fed donors.

Feeding patterns were analyzed using a BioDaq food intake monitoring system (Fig. 4). Pre-inoculation, HF feeding significantly increased meal size during the light phase (t-test, p<0.05) (Fig. 4C). There were no significant differences in meal size in the dark or meal number in the light or dark phase (Fig. 4A, G, and E). Post-inoculation, colonization with a LF microbiota normalized light phase meal size (HF-LF vs. HF-HF, One-way ANOVA, p<0.05). HF-LF animals also displayed a small reduction in meal number during the dark cycle, but this only reached significance when compared to the LF-LF groups (One-way ANOVA, p<0.05, Fig 4F). The HF-HF group still displayed a significant increase in meal size during the light phase compared to the LF groups (LF and LF-LF, One-way ANOVA, ps<0.05). There were no significant differences among groups in meal size in the dark or meal number in the light (Fig. 4B, H)

**Figure 5.**
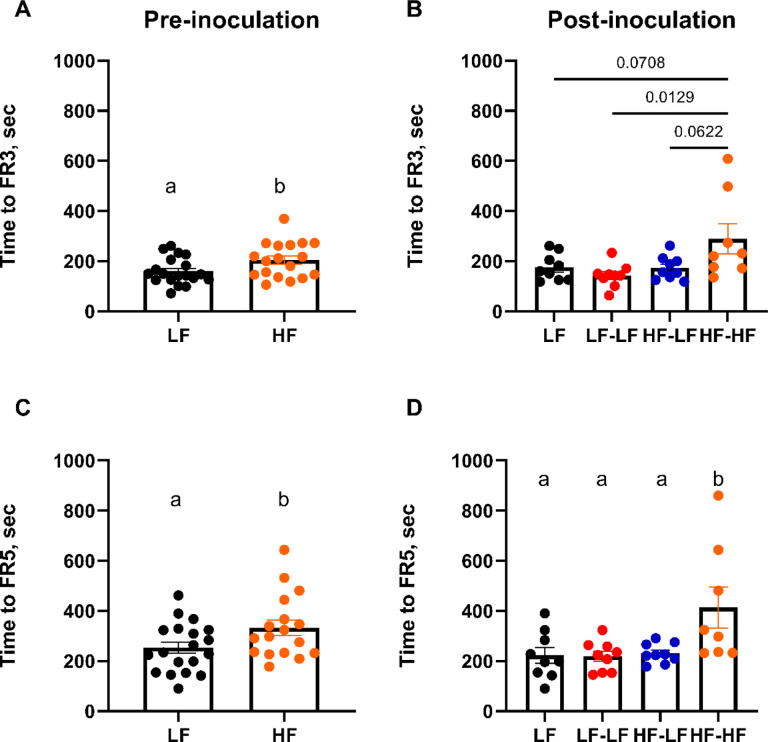
LF microbiota transfer improved acquisition time for an operant task in HF fed rats. Pre-inoculation data is shown on the left column. Post-inoculation data is shown on the right column. **A, B**. Time to acquire FR3 training criteria. **C, D**. Time to acquire FR5 training criteria. HF-HF rats showed slower acquisition learning in FR3 and FR5 (ps < 0.05). There was no difference in willingness to work for a food reward (breakpoint; **Suppl. Fig. 2**). Pre-inoculation, **LF** n=19, **HF** n=17–18. Post-inoculation, **LF (n=9)**: Low fat fed control; **LF-LF (n=9)**: Low fat rats that received microbiota from low fat fed donors; **HF-LF (n=9)**; High fat fed rats that received microbiota from low fat fed donors; **HF-HF (n=8)**: High fat fed rats that received microbiota from high fat fed donors.

A progressive ratio schedule was used to assess the animals’ drive for fat and sucrose pellets (Fig. 5). There were no differences in responses for fat versus sucrose reward pellet; thus, data were combined. Pre inoculation, LF rats achieved FR3 and FR5 criteria significantly faster (t-test, ps<0.05) than HF rats (Figs. 5A, C). Time to achieve FR criteria has been interpreted as a measure of motivation (15) as well as a measure of cognitive function and ability to learn (52, 53). There was no difference in breakpoint between LF and HF animals (**Suppl. Fig. 2A**). Post inoculation, colonization of HF fed rats with a LF microbiota (HF-LF) led to a significant improvement in task acquisition as HF-LF rats achieved FR3 and FR5 criteria as quickly as the LF animals and significantly faster than HF-HF animals (One-way ANOVA, ps<0.05). Again, there was no difference in breakpoint amongst the cohorts (**Suppl. Fig. 2B**).

### LF microbiota transfer rescued postprandial NG and NTS activation in HF fed rats.

**Figure 6.**
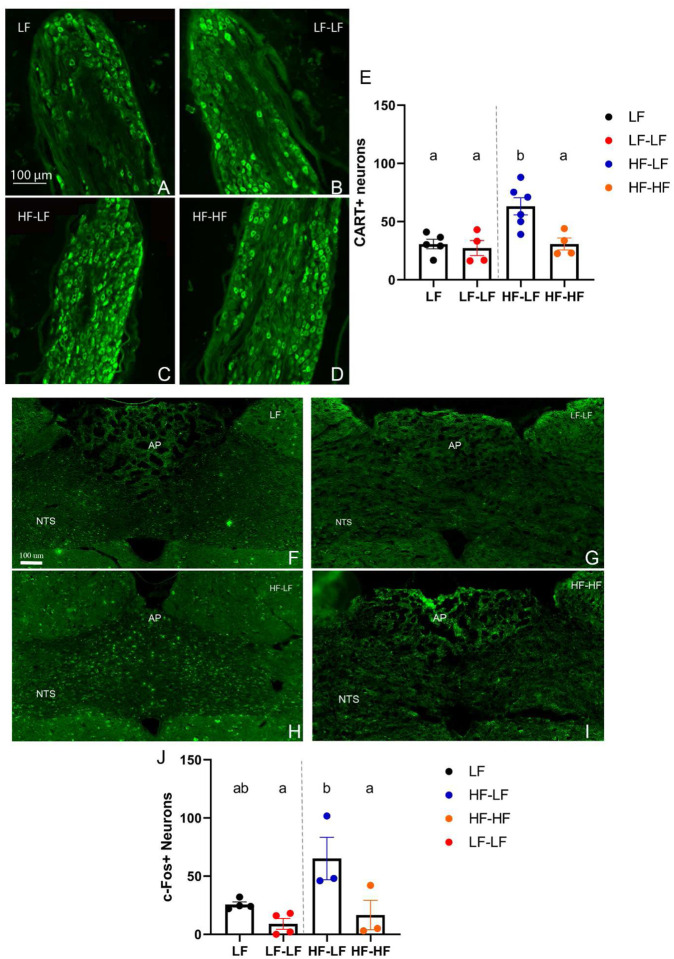
LF microbiota transfer increased post-prandial nodose ganglia (NG) and NTS activation in HF fed rats. Representative sections of NG are shown (**A-D**). Immunostaining against CART revealed that HF-LF animals had significantly higher number of CART positive neurons in the NG compared to HF-HF (p<0.05) and LF (ps<0.01) animals (**E**). Representative images of c-Fos staining in the hindbrain between bregma −13.10 and −14.10 mm are shown (**F-I**). HF-LF animals exhibited significantly higher c-Fos positive cells in the NTS compared to HF-HF (p<0.05) and LF-LF (p<0.05) rats (**J**). Bars denoted with the same letter are not statistically different. **LF (NG n=5; NTS n=4)**: Low fat fed control; **LF-LF (NG n=4; NTS n=4)**: Low fat rats that received microbiota from low fat fed donors; **HF-LF (NG n=6; NTS n=3)**; High fat fed rats that received microbiota from low fat fed donors; **HF-HF (NG n=4; NTS n=3)**: High fat fed rats that received microbiota from high fat fed donors; **AP**: Area postrema; **NTS**: Nucleus Tractus Solitarious.

Refeeding a fasted animal significantly increases CART immunoreactivity in the NG of lean (11) but not HF fed obese animals (54). Following refeeding, CART^+^ neurons were present in the NG of all animals, however there was no increase in the number of CART^+^ neurons in the HF-HF rats compared to the LF groups, despite receiving a fat-rich meal. HF-LF rats had a significantly higher number of CART positive neurons compared to all other groups (One-way ANOVA, ps<0.001) (Fig. 6A–E). Similarly, there were no differences in the number of NTS c-Fos^+^ neurons in the NTS between the HF-HF rats and the LF animals while the HF-LF animals had a significantly higher number of c-Fos^+^ neurons than LF-LF and HF-HF animals (One-way ANOVA, ps<0.05) (Fig. 6F–J).

### LF microbiota transfer decreased immune cells activation but did not affect the density of vagal afferents in the NTS in HF fed rats.

**Figure 7.**
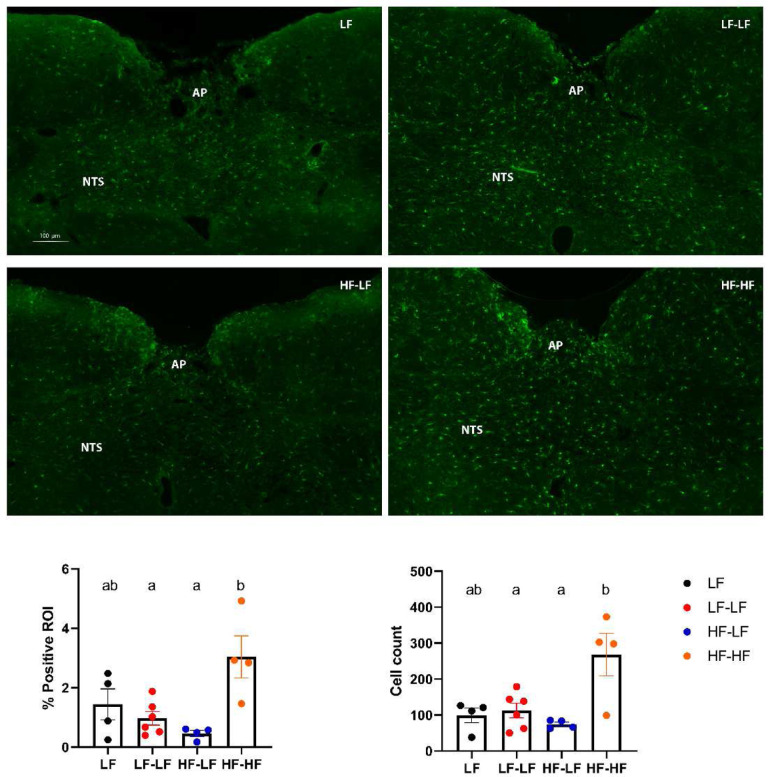
LF microbiota transfer reduced immune cells activation in the NTS in HF fed rats. Representative images of Iba-1 staining in the hindbrain between bregma −13.10 and −14.10 mm. Binary analysis of the area fraction of Iba1 immunoreactivity and cell count of microglia revealed HF fed rats that received microbiota from HF fed donors (HF-HF) had significantly higher Iba1 immunoreactivity and activated microglia than LF fed rats (LF and LF-LF) and HF fed rats that received microbiota from LF fed donors (HF-LF) (Ps < 0.05). Bars denoted with the same letter are not statistically different. **LF (n=4)**: Low fat fed control; **LF-LF (n=6)**: Low fat rats that received microbiota from low fat fed donors; **HF-LF (n=4)**; High fat fed rats that received microbiota from low fat fed donors; **HF-HF (n=4)**: High fat fed rats that received microbiota from high fat fed donors; **AP**: Area postrema; **NTS**: Nucleus Tractus Solitarious.

HF-HF animals had a significant increase in the number of Iba1^+^ cells in the NTS as well as overall positive staining compared to LF (LF and LF-LF) (One-way ANOVA, ps<0.05) (Fig. 7). Colonization of HF fed animals with a LF microbiota (HF-LF) led to a significant reduction in NTS Iba1^+^ cells and staining (HF-LF vs. HF-HF, One-way ANOVA, p<0.05). However, staining against isolectin B4 showed no difference in vagal afferent density in the intermediate NTS among the groups (**Suppl. Fig. 3)**.

## Discussion

As the prevalence of obesity and obesity-related comorbidities continues to rise worldwide, it is of primary importance to develop therapies that can aid in preventing overeating and excessive weight gain. In the current study, we sought to understand whether normalizing gut microbiota composition in HF fed obese animals would offset the detrimental physiological effects of consuming an energy dense, high in fat and sugar, diet. Prior reports (40, 55), have shown that preventing dysbiosis in HF fed animals improves metabolic outcomes. Most of these interventions were started at the same time as HF diet introduction. Here, we report that transferring the microbiota of a LF rat into an already obese HF-fed animal (HF-LF) prevented the excessive body weight gain characteristically observed with HF feeding, normalized meal patterns, and improved vagally mediated gut-brain axis signaling and learning.

Chronic consumption of a HF diet significantly decreases bacterial diversity and alters the gut microbiota profile. Consistent with existing data, our results show that HF animals had significantly lower bacterial diversity than LF animals (12). HF animals recolonized with a HF microbiota (HF-HF) also displayed the hallmarks of HF feeding (56), including increased Firmicutes abundance, in particular *Dorea* (40) and reduced Bacteroidetes. Conversely, HF animals that received LF microbiota (HF-LF) had an increase in the relative abundance of bacteria belonging to the Bacteroidetes phylum. It is possible that members of the Bacteroidetes phylum are playing a role in the reduced weight gain observed in these animals as it has been previously shown that body weight loss is positively associated with increased Bacteroidetes abundance in obese human subjects (57). Additionally, HF-LF animals did not display the typical increase in Firmicutes abundance that has been associated with HF feeding and obesity (14). Firmicutes have been hypothesized to be more efficient at extracting energy from food promoting weight gain (7, 58, 59). Thus, by transferring lean microbiota into HF-fed rats, we may have counteracted the establishment of these bacteria and prevented the excessive weight gain typically induced by HF feeding.

Although a LF microbiota transfer into a HF animal did not rescue bacterial diversity, the microbiota profile of these animals resembles more closely that of LF animals than HF animals. LF microbiota transplant to HF animals resulted in normalized – reduced - abundance levels, comparable to LF animals, of members of the *Lacnospiraceae* (*Dorea* and *Producta*) and *Clostridiaceae* (*SMB53* and *Celatum*) families of the Firmicutes phylum. This validates the establishment of lean microbiota in HF fed animals since our data and previous studies have shown that HF diet consumption increases the abundance of these bacteria compared to LF feeding (12, 37). In addition, lower abundances of these taxa have been associated with reduced SCFA fecal, reduced adiposity and gut permeability, and improved cardiometabolic health profile (59). In contrast, the relative abundances of several members of this phylum were not affected by the microbiota transfer. The taxa *Oscillospira,* associated with lower BMI (60) and reduction of body weight (61), was found in similar abundance levels in HF-LF and HF-HF animals. This is likely due to increased bile secretion in response to the HF diet as it has been shown that HF diet consumption increases bile secretion (62) and there is a positive correlation between bile secretion and *Oscillospira* abundance level (63). Belonging to the *Ruminococcaceae* family, *Ruminococcus* were found to be significantly depleted in HF compared to LF animals. These lack of the *Ruminococcaceae* family might be associated with suboptimal gut health in HF fed animals (64).

In rats, chronic HF feeding triggers an increase in caloric intake and leads to body weight gain over time. Consistent with these reports, our results show that HF animals had a significantly higher caloric intake and gained significantly more weight than LF animals pre-inoculation. The increase in intake was driven by an increase in meal size, especially during the light cycle. (65). Meal size during the light phase of the light cycle was significantly larger in HF animals. While some effect of HF feeding on meal size may be driven by the novelty and increased palatability of the diet, HF rats that received HF microbiota still displayed increased meal size after being on the diet over a prolonged period of time. Thus, meal pattern disruptions elicited by a HF diet are long-lasting and have been linked to increased body fat accumulation in animal models (66) and human subjects (67). However, normalizing the gut microbiota composition can rescue normal feeding patterns since HF rats that received LF microbiota did not exhibit increased meal size despite being on a HF diet. It is noteworthy to mention that these rats did exhibit increased meal size prior to the microbiota transfer. Overall, HF animals that received LF microbiota transfer (HF-LF) consumed significantly less calories and gained significantly less body weight than HF animals that received HF microbiota (HF-HF). Male Wistar rats pair-fed a HF or HS (high sucrose) diet gained significantly more body weight than their LF counterparts (68–70). Similarly, male Sprague-Dawley rats maintained on a HF (45% fat) diet for over six months gain significantly more body weight and fat mass than LF animals (38). Here we show that altering the gut microbiota composition to a lean profile counteracts the obesogenic effect of a HF diet independent of dietary changes. To our knowledge, this is the first time these findings have been reported in conventional animals fed a HF diet.

Post-prandial gut-brain communication plays a key role in regulating satiation and meal termination (56, 71–73). Previous studies from our lab have demonstrated that HF feeding affects the gut-brain vagal system structure and function, somewhat independently of one another. HF diet consumption affects the density of vagal terminals in the NTS and that these effects are dependent both upon length of time on the diet and diet composition (12, 13). One week of a 60% HF diet exposure led to a transient decrease in vagal fibers in the NTS followed by sprouting, noticeable three weeks on the diet (13). In contrast, we have observed significant reduction in vagal fiber density in the NTS after four weeks and 8 weeks of 45% HF diet exposure (12, 37, 40). Taken together, these results suggest that the morphological reorganization of the gut-brain vagal axis observed in response to a HF diet fluctuates over time and stabilizes. Sprouting following initial withdrawal does not appear to restore function, as rats fed a HF diet remained insensitive to gut satiety peptides (13, 39). Consistent with these findings, our results show no significant difference in the density of vagal afferents in the intermediate NTS among the groups. It is likely that since these animals have been on HF diet for over six months, there was an initial decrease in the density of VAN when the diet was first introduced followed by sprouting. Restoration of structure in the HF-HF animals did not translate in re-establishment of function.

Gut-brain mediated satiation signaling is the major determinant of meal size GI satiety peptides, such as CCK and GLP-1 activate vagal to trigger meal termination (74, 75). CART neuropeptide expression is induced in the NG upon feeding, and this response is mediated by CCK (21, 22). CART and CCK-mediated vagal signaling is further associated with c-Fos expression, a marker of neuronal activation, in the NTS and other central areas involved in the control of food intake (23, 54). These satiation and satiety signals are known to be compromised, i.e., blunted, in HF feeding and obesity as a result of decreased sensitivity to CCK (10). Post-prandial vagal activation is dependent on the amount of kcal consumed as well as macronutrient makeup, with fat consumption leading to greater activation, for the same amount of kcal, than carbohydrates and proteins (76). There were no differences in the number of CART^+^ neurons in the NG and c-Fos^+^ neurons in the NTS following refeeding between HF-HF rats and LF animals, despite the HF-HF rats consuming a fat rich meal. Colonization of HF animals with a LF microbiota resulted in a significantly greater number of CART^+^ neurons in the NG and c-Fos^+^ neurons in the NTS after refeeding compared to all other groups. This supports our hypothesis that normalizing the gut microbiota improves vagal signaling independent of diet since HF-LF animals are exhibiting greater neuronal activation following a meal than HF-HF rats.

Diet and microbiota-driven alteration in vagal signaling are accompanied by an increase presence of Iba1^+^ positive immune cells along the gut-brain axis (12, 13, 38, 40, 77, 78). Recruitment of these cells appears to be necessary for diet-driven alterations of vagal signaling as their pharmacological inhibition prevents diet-associated vagal remodeling and hyperphagia (13). Similarly to what had previously been reported (13, 37, 38, 40), we observed an increased in the number of Iba1^+^ cells (and overall staining) in the NTS of HF-HF rats. Conversely, HF animals that received LF microbiota do not exhibit an increase in inflammatory markers (Iba1^+^ cells) in the intermediate NTS, showing that changes in microbiota composition are sufficient to decrease immune cells recruitment along the gut-brain axis, independently of diet. This reduction in neuroinflammation may mediate the improved post-prandial neuronal response.

In addition to its classic role in homeostatic regulation of feeding, gut-brain has recently been shown to modulate food-driven reward. A recent study unveiled a direct, multi-synaptic pathway between the gut and the dopaminergic reward system. Han *et al*. showed that right NG vagal sensory fibers that innervate the gut project to the ventromedial NTS. These cells in turn project to the medial Parabrachial Nucleus, which then targets the Substantia nigra. Activation of right vagus-parabrachial-nigrostriatal pathway is required for nutrient sensing, sustained self-stimulation, and stimulus preference (36). This gut to reward pathway is blunted in obesity and diet-induced changes in gut microbiota are sufficient to induce deficits in vagally-mediated reward (15). Progressive ratio (PR) responding has been widely used to study willingness to work for a natural reward or addictive substance in animal subjects. In recent years, studies have further shown that this task can also be used to evaluate learning, memory, and cognitive function (52, 53). In this task, animals are trained to emit a response to obtain a reward. Thus, acquisition time for the task can be reflective of learning capabilities. Breakpoint, which is the maximum number of responses the animal emits, relates to the amount of “work” a subject is willing to perform to obtain a reward. Consistent with a reduced food-driven reward, HF feeding has been shown to reduce motivation to work for a palatable reward (79, 80). In a recent study from our lab, we showed that HF rats exhibit significantly longer acquisition times for a fixed ratio (FR) 3 schedule compared to LF animals, but no difference in breakpoint. We further showed that colonization of GF rats with HF microbiota significantly lowered breakpoint and increased task acquisition time. These behaviors were directly linked to downregulation of the dopaminergic reward system as a result of vagal signaling alterations in response to changes in the microbiota composition (15). Consistent with these findings, HF feeding significantly increased the time to achieve FR3 and FR5, but had no effect on breakpoint. LF microbiota transfer to HF rats significantly decreased FR3 and FR5 acquisition time. Vazquez *et. al*. used acquisition time for a fixed ratio schedule task to assess long-term potentiation and memory, and determined that intact gut-brain communication through the vagus nerve improves learning and memory in rats (53). This data further supports our hypothesis that normalizing the gut microbiota in HF animals restores gut-brain vagal signaling.

In conclusion, this study provides a proof of concept that changing the gut microbiota composition in diet-induced obesity can improve many of the detrimental consequences of chronic high fat-high sugar diet consumption. There was a symbiotic effect of transferring lean microbiota as a probiotic and providing inulin as a prebiotic to HF-fed, obese animals that resulted in a beneficial shift in the microbiota composition even when the animals remained on a HF diet. It is worth nothing that while the present study design does not allow us to distinguish between the effects of FMT and inulin or the synergistic actions of both, it has been previously shown that inulin does not affect body weight and adiposity in LF-fed mice (81). Thus, the effects of inulin are mediated via microbiota changes, which in turn alters nutrient metabolism. HF animals that receive LF microbiota gained less body weight, exhibited normalized feeding patterns, improved memory and cognition, and had restored function and structure of the gut-brain vagal signaling pathway. These data further emphasize the importance of the gut microbiota population in the regulation of feeding behaviors and the development of obesity, thus representing a potential therapeutic target to prevent obesity and its comorbidities.

## Data Availability

All data supporting the findings of this study are available within the paper and its Supplementary Information. Raw data files will be made available upon request.
